# FAD-deficient P187S mutation of NAD(P)H:quinone oxidoreductase 1 (NQO1*2) binds and accelerates β-amyloid aggregation

**DOI:** 10.1042/BSR20220643

**Published:** 2022-11-14

**Authors:** Sudipta Panja, David Siegel, Simonetta Camandola, Rafael de Cabo, David Ross, Krishna M.G. Mallela

**Affiliations:** 1 Department of Pharmaceutical Sciences, Skaggs School of Pharmacy and Pharmaceutical Sciences, University of Colorado Anschutz Medical Campus, 12850 East Montview Boulevard, MS C238-V20, Aurora, CO 80045, U.S.A.; 2 Experimental Gerontology Section, National Institute of Aging, National Institutes of Health, Baltimore MD, U.S.A.

**Keywords:** Alzheimers disease, binding, Biophysics, fluorescence, kinetics, NQO1

## Abstract

Alzheimer’s disease (AD) is one of the most prominent neurodegenerative diseases. Results from animal and cellular models suggest that FAD-deficient forms of NAD(P)H quinone oxidoreductase 1 (NQO1) may accelerate the aggregation of Alzheimer’s amyloid-β peptide (Aβ_1-42_). Here, we examined *in vitro* whether NQO1 and its FAD-deficient P187S mutation (NQO1*2) directly interact with Aβ_1-42_ and modify its rate of aggregation. When monitored using the fluorescence of either noncovalent thioflavin T (ThT) or HiLyte Fluor 647 (HF647) dye covalently attached to the Aβ_1-42_ peptide, the aggregation kinetics of Aβ_1-42_ were markedly more rapid in the presence of NQO1*2 than the wild-type (WT) NQO1. Experiments using apo-NQO1 indicate that this increase is linked to the inability of NQO1*2 to bind to FAD. Furthermore, dicoumarol, an NQO1 inhibitor that binds near the FAD-binding site and stabilizes NQO1*2, markedly decreased the aggregation kinetics of Aβ_1-42_. Imaging flow cytometry confirmed *in-vitro* coaggregation of NQO1 isoforms and Aβ_1-42_. Aβ_1-42_ alone forms rod-shaped fibril structures while in the presence of NQO1 isoforms, Aβ_1-42_ is incorporated in the middle of larger globular protein aggregates surrounded by NQO1 molecules. Isothermal titration calorimetry (ITC) analysis indicates that Aβ_1-42_ interacts with NQO1 isoforms with a specific stoichiometry through a hydrophobic interaction with positive enthalpy and entropy changes. These data define the kinetics, mechanism, and shape of coaggregates of Aβ_1-42_ and NQO1 isoforms and the potential relevance of FAD-deficient forms of NQO1 for amyloid aggregation diseases.

## Introduction

NAD(P)H quinone oxidoreductase 1 (NQO1) is a two-electron reductase, which has diverse cellular functions and is known to play critical roles in protection against oxidative stress and in redox control via multiple mechanisms [1,2]. These include generation of antioxidant forms of CoQ10 and vitamin E, a superoxide reductase activity and as a vital component of the plasma membrane redox system (PMRS). NQO1 has also been reported to protect multiple proteins against proteasomal degradation, play a role in mRNA translation, associate with microtubules during cell division, generate NAD^+^, and interact with sirtuins [3]. Changes in pyridine nucleotide redox ratios result in a conformational change in NQO1, which governs interactions with NQO1 antibodies and potentially other proteins suggesting it may also play a role as a redox switch [2,3].

**Figure 1:**
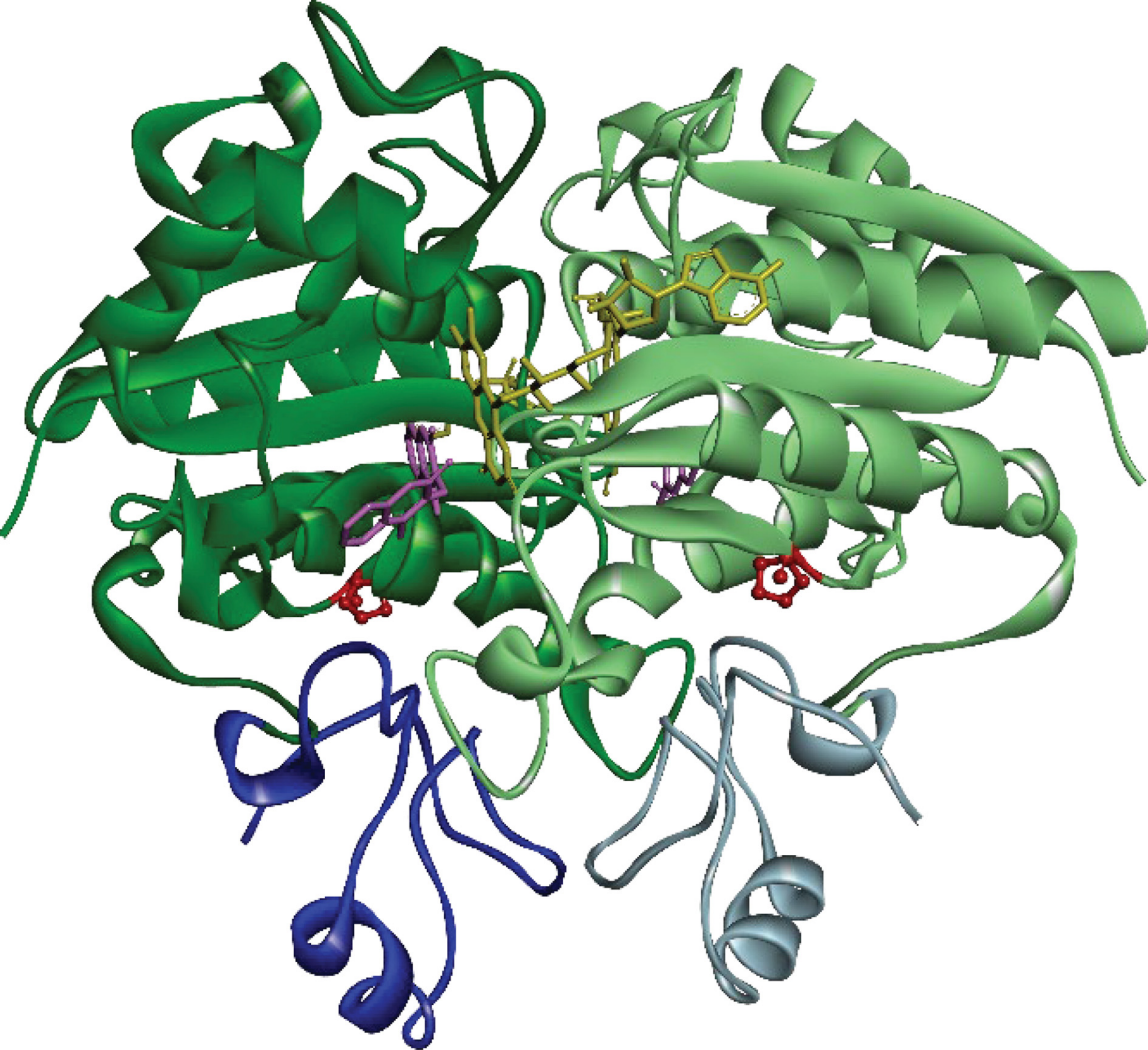
Crystal structure of the dimeric form of WT-NQO1 in complex with its substrate flavin adenine dinucleotide (FAD; yellow) and its inhibitor dicoumarol (light purple) (PDB ID 2F1O) Green colors show N-terminal domains (NTD) and blue colors show C-terminal domain (CTD). Dark and light colors represent the different monomers in the NQO1 dimer. The NQO1*2 protein has the P187S mutation. The proline residues at position 187, one in each monomer, are shown in red and are at the interface of the NTD and CTD.

Oxidative stress has been associated with the progression of Alzheimer’s disease (AD) [4] and the potential links between oxidative stress, NQO1 and AD have been reviewed [5]. NQO1 is elevated in AD brains relative to normal brains, is observed in neurons with either mature neurofibrillary tangles or preneurofibrillary lesions in AD but not from age-matched control brains [6,7] and is also localized around senile plaques [8]. The P187S mutation in wild-type (WT) NQO1, known as NQO1*2, is more prevalent in Asian populations, and the NQO1*2 genotype was found to be associated with AD in a Chinese population [9]. The structure of NQO1 highlighting the location of the P187S mutation is shown in Figure 1. Amyloid-β peptide (Aβ_1-42_) has been associated with driving aberrant protein aggregation and the generation of plaques in AD [10,11]. Mutations in amyloid precursor protein and presenilin 1 and 2 that account for the majority of early onset familial AD increase the production of Aβ_1-42_ [12,13]. NQO1 and cytochrome b5 reductase are important components of the antioxidant PMRS and neurons overexpressing these two enzymes exhibited increased resistance to amyloid-β peptide [14].

The flavoproteome consists of approximately 100 proteins and riboflavin deficiency destabilizes many flavoproteins due to a lack of FAD, resulting in protein degradation largely via the proteasome. Accumulation of unstable proteins as a result of a lack of FAD has been suggested to overload the cellular protein quality control system leading to protein aggregation [2,15]. This mechanism was explored in more detail using NQO1 as a model flavoprotein. The NQO1*2 protein has a much lower affinity for FAD relative to the WT NQO1 protein [16,17]. FAD-deficient NQO1 isoforms either as a result of riboflavin-deficiency or the NQO1*2 polymorphism coaggregated with Aβ_1-42_ [18]. The implication from these earlier animal and cellular studies was that coaggregation of FAD-deficient forms of NQO1 could increase amyloidogenesis and potentially contribute to protein aggregation diseases.

Coaggregation of NQO1 and Aβ_1-42_ in animals and cellular models does not prove a direct interaction between the two proteins. There can be a third protein or an alternate pathway that can allow both proteins to coaggregate without direct interaction. In this work, we have examined whether Aβ_1-42_ specifically interacts with NQO1 isoforms *in vitro* in the absence of any other proteins. We probed the kinetics of Aβ_1-42_ aggregation, the nature of Aβ_1-42_ interaction with NQO1 isoforms, and the type of protein aggregates formed during the coaggregation of Aβ_1-42_ with both FAD-deficient forms of NQO1 (apo-NQO1 and NQO1*2) and FAD-competent WT-NQO1. Our data describe the increased rate of Aβ_1-42_ aggregation with NQO1*2 relative to WT-NQO1 and for the first time show the shape and size of protein aggregates of Aβ_1-42_ with different forms of NQO1.

## Materials and methods

### Materials

Human Aβ_1-42_ as a click peptide was purchased from GenScript (Catalog# RP10017) and used as received. The click peptide undergoes a chemical reaction at physiological pH leading to the formation of monomeric Aβ_1-42_ [19]. Thioflavin T (ThT), FAD, nicotinamide adenine dinucleotide-reduced form (NADH), and dicoumarol were purchased from Sigma-Aldrich. Aβ_1-42_ covalently linked to HiLyte Fluor 647 (HF647) dye was purchased from AnaSpec (Catalog# AS-64161). Ultrapure Milli Q water was used throughout the study. Aggregation studies were performed in 100 mM potassium phosphate buffer, pH 7.4. Recombinant human WT NQO1 and the NQO1*2 variant were purified from *Escherichia coli* using Cibacron-blue affinity chromatography as previously described [20,21]. The purified proteins resolved as single bands near 30 kDa. FAD-deficient apo NQO1 was generated by incubating WT NQO1 in two cycles of 50 mM potassium phosphate buffer, pH 7.4 containing 2 M potassium bromide as previously described [22]. Purified NQO1 proteins were stored in 50 mM potassium phosphate buffer, pH 7.4 containing 250 mM sucrose and 5 µM FAD at –80°C.

### Sample preparation

A stock solution of Aβ_1-42_ click peptide was prepared by dissolving the peptide in 0.1% trifluoroacetic acid aqueous solution (v/v) at 4°C. To calculate the exact concentration, we used an extinction coefficient of 1490 M^−1^ cm^−1^ at 280 nm [23]. The lyophilized HF647-labeled human Aβ_1-42_ was dissolved in 1% ammonium hydroxide (v/v) at 4°C. The concentration of the peptides in the aliquots was determined using extinction coefficient of 250000 M^−1^ cm^−1^ at 649 nm for the HF647 dye [24]. Aβ_1-42_ solutions were vortexed and centrifuged to remove any larger particles. Aβ_1-42_ stock sample was divided into aliquots and stored at –80°C until further use. A fresh aliquot was used for each experiment to avoid repeated freeze-thaw cycles that could trigger potential aggregate formation [24]. For the HF647 fluorescence assay, the samples contained 98% unlabeled Aβ_1-42_ and 2% Aβ_1-42_ covalently tagged with HF647. Dicoumarol stock solutions were prepared in aqueous solution of 0.1 N NaOH. For anaerobic experiments, reduced NQO1 (NQO1 plus 1 mM NADH) stock was diluted immediately before the experiment into a buffer containing 300 μM NADH. Solutions were purged with excess N_2_ for 1–2 h in a cuvette, and the cuvette was sealed prior to the experiments. NQO1 remained in its reduced state at the end of the experiment as evident from its characteristic absorption spectrum.

### Absorption spectroscopy

UV–vis absorption spectra were recorded on an Agilent 8453 UV–visible absorption spectrophotometer equipped with a temperature-controlled cell holder using a 1 cm pathlength cuvette and the appropriate solvent blank.

### Fluorescence spectroscopy

Steady-state fluorescence spectra were recorded using a PTI Quantamaster fluorimeter equipped with temperature-controlled water-cooled cuvette holder. The samples were excited at 440 nm for ThT and the fluorescence emission was recorded from 450 to 600 nm. To acquire the fluorescence spectra of HF647, samples were excited at 600 nm and emission spectra were recorded from 620 to 760 nm. A 1 cm path-length quartz cuvette was used for these experiments. Aβ_1-42_ kinetics data of intensity count were normalized from 0 to 1. NQO1 and NQO1*2 self-aggregation data were normalized with respect to Aβ_1-42_ kinetics intensity counts. All kinetic data shown in this manuscript were representative of triplicate experiments.

### Fluorescence flow cytometry

Samples were subjected to flow cytometry analysis using a 12 channel Amnis® ImageStream®X Mark II (Luminex) imaging flow cytometer (Rocky Mountain VA Flow Cytometry Core). Samples were acquired at 60× magnification using INSPIRE (Millipore Sigma, Seattle, WA) software [25,26]. Aggregates were imaged using both brightfield (BF) LED and the 642 nm laser set to 15 mW to excite the HF647 dye. All data were saved as raw image files and analyzed using IDEAS v6.2 software. FlowCam was used to determine the number of particles in solutions. Statistically significant differences between the groups were analyzed by one-way ANOVA using GraphPad Prism 7 software (San Diego, CA).

### Isothermal titration calorimetry

Isothermal titration calorimetry (ITC) experiments were conducted using MicroCal PEAQ-ITC (Malvern, UK) at 37°C. NQO1 in 50 mM sodium phosphate was titrated with Aβ_1-42_ from a syringe (130 μM). These experiments were conducted using the Aβ_1-42_ monomer by dissolving the click peptide in phosphate buffer, which does not form amyloid fibrils without shaking at 20°C for at least 1 day. Except for the first injection (0.2 μL), the volumes for the remaining eighteen injections were 2 μL each. Each injection duration was 4 s and spacing between individual injections was 150 s. Blank ITC thermograms (titration of Aβ_1-42_ into same phosphate buffer without NQO1) recorded under identical conditions were subtracted point-by-point from Aβ_1-42_-NQO1 binding thermograms. Data were analyzed using MicroCal PEAQ-ITC analysis software provided with the instrument. Respective blank-subtracted curves were fitted using a single set multiple site model to calculate the dissociation constant (K_d_), binding stoichiometry (N), and thermodynamic enthalpy (ΔH). Thermodynamic entropy (ΔS) values were calculated from these fit parameters and the error propagation formulae [27]. The titration data shown were representative of triplicate experiments.

## Results

### Aβ_1-42_ aggregation kinetics is accelerated in the presence of NQO1*2 isoform

**Figure 2:**
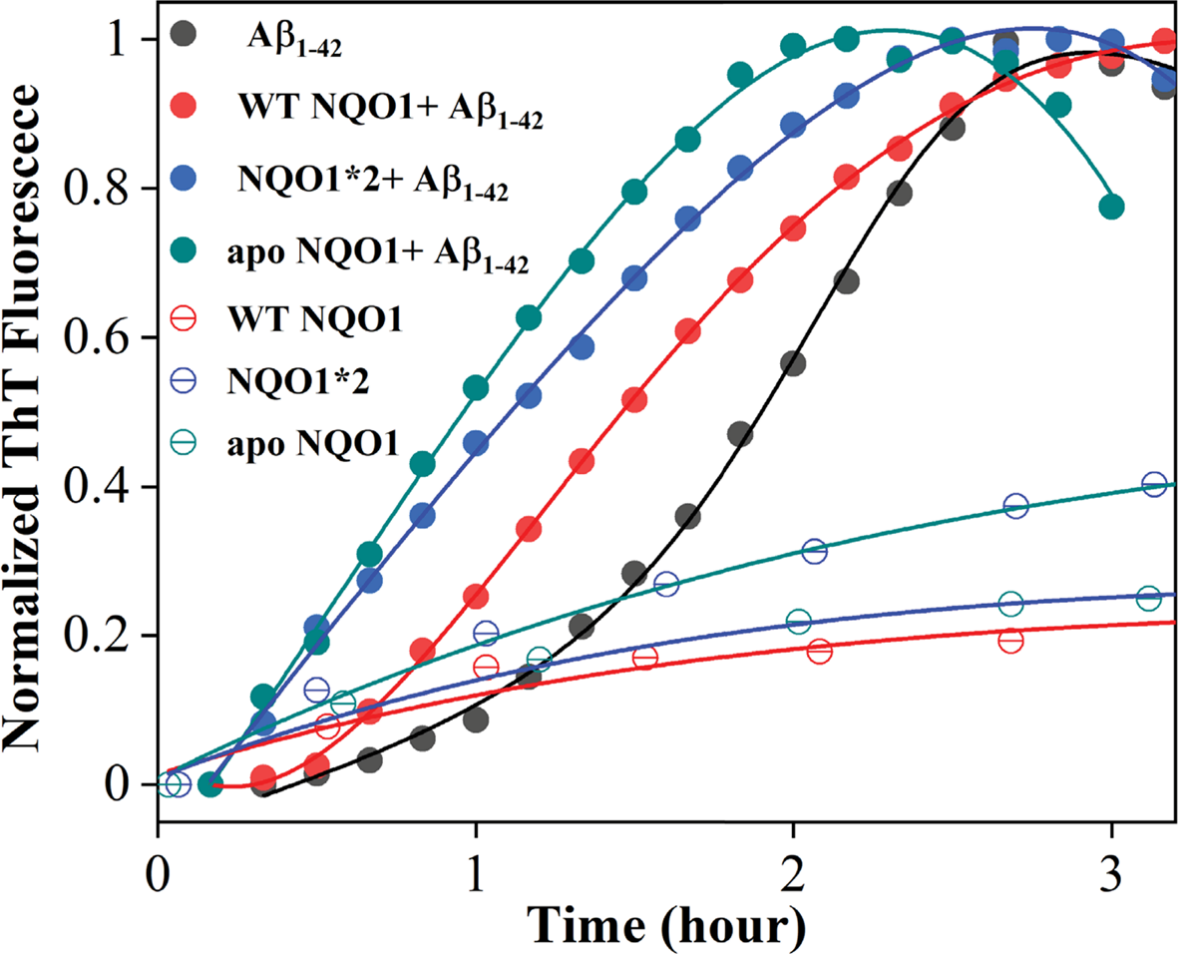
ThT (20 μM) fluorescence assay of Aβ_1-42_ (10 μM) aggregation kinetics in the absence and presence of 10 μM of WT-NQO1, NQO1*2, and apo-NQO1 in 50 mM phosphate buffer, pH 7.4 at 37°C under atmospheric conditions Note that the downward curvature at longer incubation times corresponds to aggregates settling to the bottom of the cuvette.

Fluorescence is a sensitive tool to determine the change in the local environment of fluorophores. ThT fluorescence increases upon binding to amyloid fibrils [28,29], and was used to characterize the aggregation of Aβ_1-42_ in the presence of various NQO1 isoforms (Figure 2). Aggregation of Aβ_1-42_ in general undergoes a stochastic polymerization reaction with a lag phase corresponding to the nucleation (formation of oligomers) and a growth phase corresponding to the propagation of the amyloid nuclei [30,31]. Under continuous agitation at 37°C and at pH 7.4, Aβ_1-42_ peptide took 3 h to form ThT-responsive amyloid aggregates, which showed a characteristic prominent lag phase (black curve in Figure 2). When the same aggregation assay was performed in the presence of NQO1*2, Aβ_1-42_ aggregation occurred at a faster rate (blue curve with solid symbols in Figure 2) than in the presence of WT-NQO1 (red curve with solid symbols) or Aβ_1-42_ alone (black curve). Earlier studies have shown that NQO1*2 binds to FAD with ∼400-fold lesser affinity than that of the WT-NQO1 [17]. To confirm that the NQO1*2 acceleration of Aβ_1-42_ aggregation kinetics is because of its inability to bind to FAD, the apo-form of WT-NQO1 that completely lacks FAD was used as a control. The aggregation kinetics of Aβ_1-42_ in the presence of apo-NQO1 (green curve with solid symbols in Figure 2) were much faster than those observed with WT-NQO1 (red curve) or NQO1*2 (blue curve), indicating that FAD-deficient NQO1 isoforms accelerate Aβ_1-42_ aggregation kinetics. Amyloid aggregation kinetics with NQO1*2 or apo-NQO1 has a shorter lag phase (<5 min) compared with WT-NQO1, which has a prominent lag phase of ∼0.5 h. The different kinetic patterns of Aβ_1-42_ aggregation for various NQO1 isoforms (Aβ_1-42_ alone < WT-NQO1 < NQO1*2 < apo-NQO1; Figure 2) follow the order of the lack of bound FAD, indicating that the FAD-deficiency leads to NQO1-induced acceleration of Aβ_1-42_ aggregation kinetics. Although amyloid aggregation has been known to be stochastic in kinetics [30], three independent measurements showed a similar trend. Subsequent studies focused on the prevalent cellular NQO1 isoforms, NQO1*2 and WT- NQO1.

**Figure 3:**
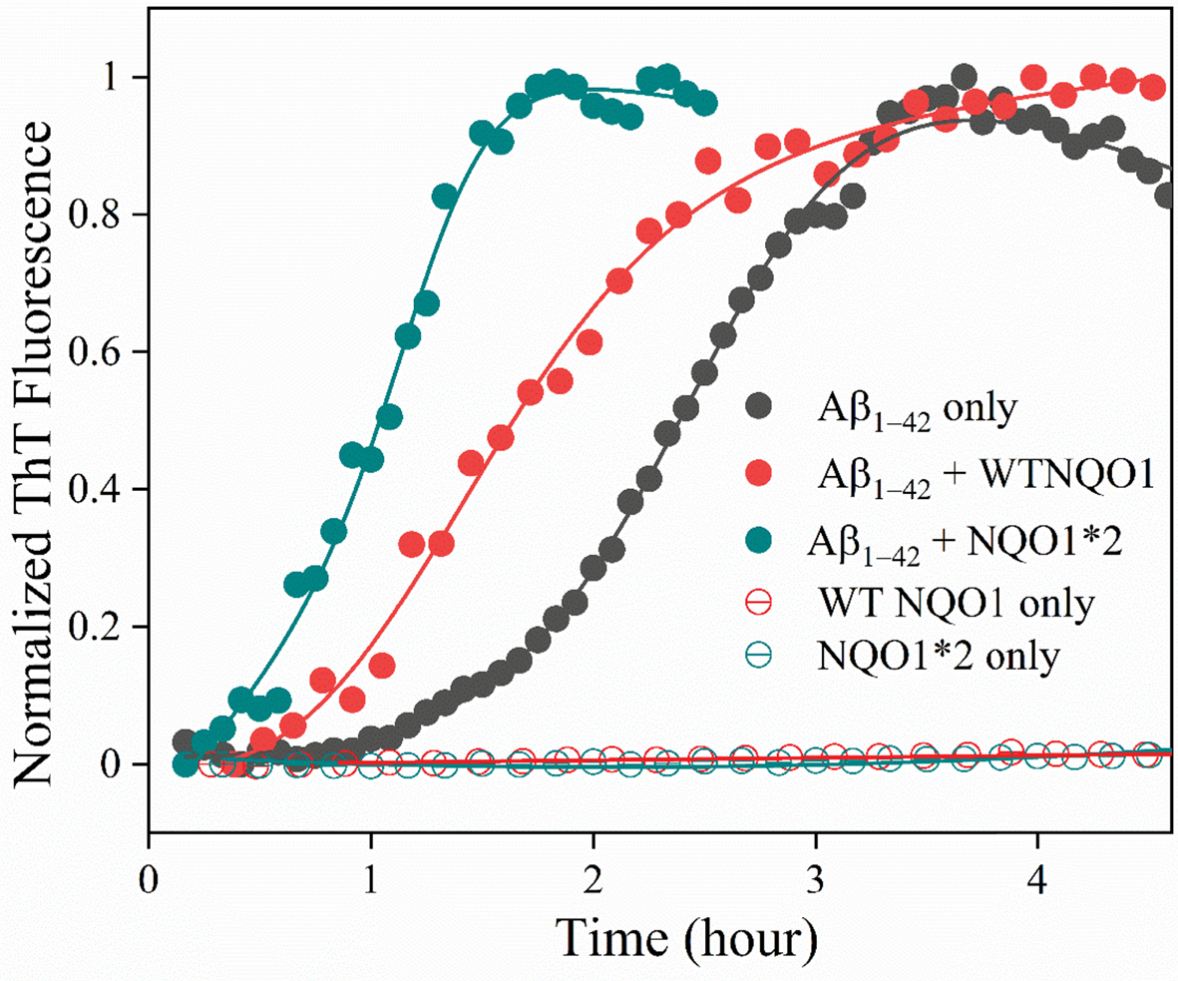
ThT (20 PM) fluorescence assay of Aβ_1-42_ (10 μM) aggregation kinetics in the absence and the presence of NQO1*2 (10 μM) in 50 mM phosphate buffer, pH 7.4 at 37°C under anaerobic conditions WT-NQO1 and NQO1*2 in the absence of Aβ_1-42_ did not show any aggregation.

The kinetics of aggregation in Figure 2 were observed under aerobic conditions, where reduced NQO1 slowly oxidizes leading to a destabilized form, which then slowly self-aggregates (see open symbols, Figure 2). Although the kinetics of NQO1 self-aggregation are markedly slower compared with Aβ_1-42_ kinetics, it is quite possible that the presence of preformed NQO1 aggregates may act as nucleation seeds for Aβ_1-42_ aggregation. To exclude this possibility, we performed the Aβ_1-42_ aggregation assay under anaerobic conditions (extensive degassing of solution with N_2_ and sealing the cuvette in the presence of N_2_ to prevent oxidation of NQO1) for WT-NQO1 and NQO1*2 (Figure 3). As expected, under anaerobic conditions, no visible aggregates were observed for WT-NQO1 and NQO1*2 (open symbols in Figure 3). However, under identical conditions, Aβ_1-42_ polymerization kinetics were still accelerated by the presence of NQO1*2 (green curve in Figure 3) compared with WT-NQO1 (red curve in Figure 3) or Aβ_1-42_ alone (black curve in Figure 3). These results exclude the possibility that NQO1 aggregates were acting as nucleation seeds for Aβ_1-42_ aggregation. Furthermore, the data suggest that the accelerated polymerization is likely due to a direct interaction between monomeric NQO1 and Aβ_1-42_, which was further confirmed by ITC binding (below).

**Figure 4:**
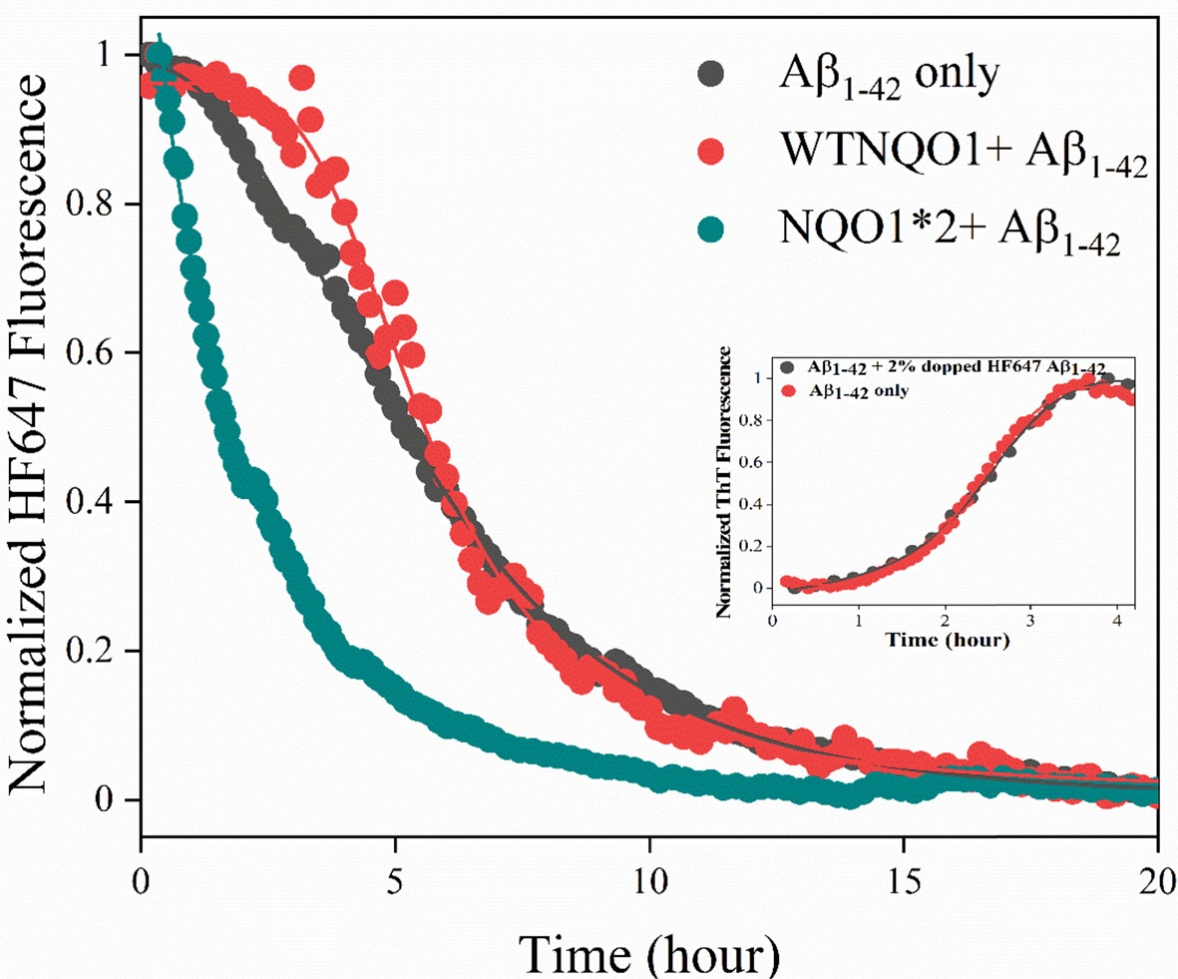
Aggregation kinetics of Aβ_1-42_ (10 μM) spiked with 2% of HF647 covalently attached (λ_ex_ = 600 nm, λ_em_ = 665 nm) in 50 mM sodium phosphate buffer, pH 7.4 as a function of incubation time in the absence and presence of WT-NQO1 (10 μM) and NQO1*2 (10 μM) at 37°C under atmospheric conditions Inset shows no effect of spiking with 2% Aβ_1-42_-HF647 on Aβ_1-42_ aggregation kinetics monitored using ThT fluorescence.

As shown in Figure 2, ThT is not able to distinguish between Aβ_1-42_ aggregates and NQO1 aggregates as its fluorescence increases even when it binds to NQO1-only aggregates. To obtain specific information regarding Aβ_1-42_ aggregation kinetics that is distinct from NQO1 aggregation, experiments were performed using Aβ_1-42_ peptide covalently labeled with HF647 dye [24]. HF647 is a solvatochromic dye whose fluorescence is sensitive to the micropolarity of the fluorophore, and its fluorescence decreases with amyloid formation [24]. Doping the Aβ_1-42_ sample with 5% of Aβ_1-42_ labeled with HF647 has been shown earlier to have no effect on the amyloid aggregation kinetics [32], and our samples contain only 2% Aβ_1-42_ labeled with HF647. Figure 4 shows the Aβ_1-42_ aggregation kinetics in the absence and presence of NQO1 isoforms when monitored using the HF647 fluorescence. Inset shows that doping with 2% Aβ_1-42_ labeled with HF647 did not affect the amyloid aggregation kinetics. The kinetic traces of Aβ_1-42_ alone (black curve in Figure 4) showed a decrease in fluorescence signal with time indicating the formation of mature amyloid fibrils, and it took about 15–20 h for the aggregation to complete. The Aβ_1-42_ aggregation kinetics were much faster (within 5 h) in the presence of NQO1*2 (green curve in Figure 4). In the case of WT-NQO1 (red curve, Figure 4), Aβ_1-42_ aggregation kinetics followed a similar trend as that of Aβ_1-42_ alone. These results confirmed that FAD-deficient NQO1*2 significantly accelerated Aβ_1-42_ aggregation, consistent with kinetics monitored by the noncovalent fluorescent dye ThT (Figure 2). It is important to note that ThT detects smaller size aggregates, whereas HF647 detects mature amyloid fibrils as evident from the respective kinetics (Figures 2 and 4); and hence, the kinetics detected by both fluorescent probes represent two distinct phases of Aβ_1-42_ aggregation.

### Dicoumarol inhibits the Aβ_1-42_ aggregation kinetics accelerated by FAD-deficient NQO1*2

**Figure 5:**
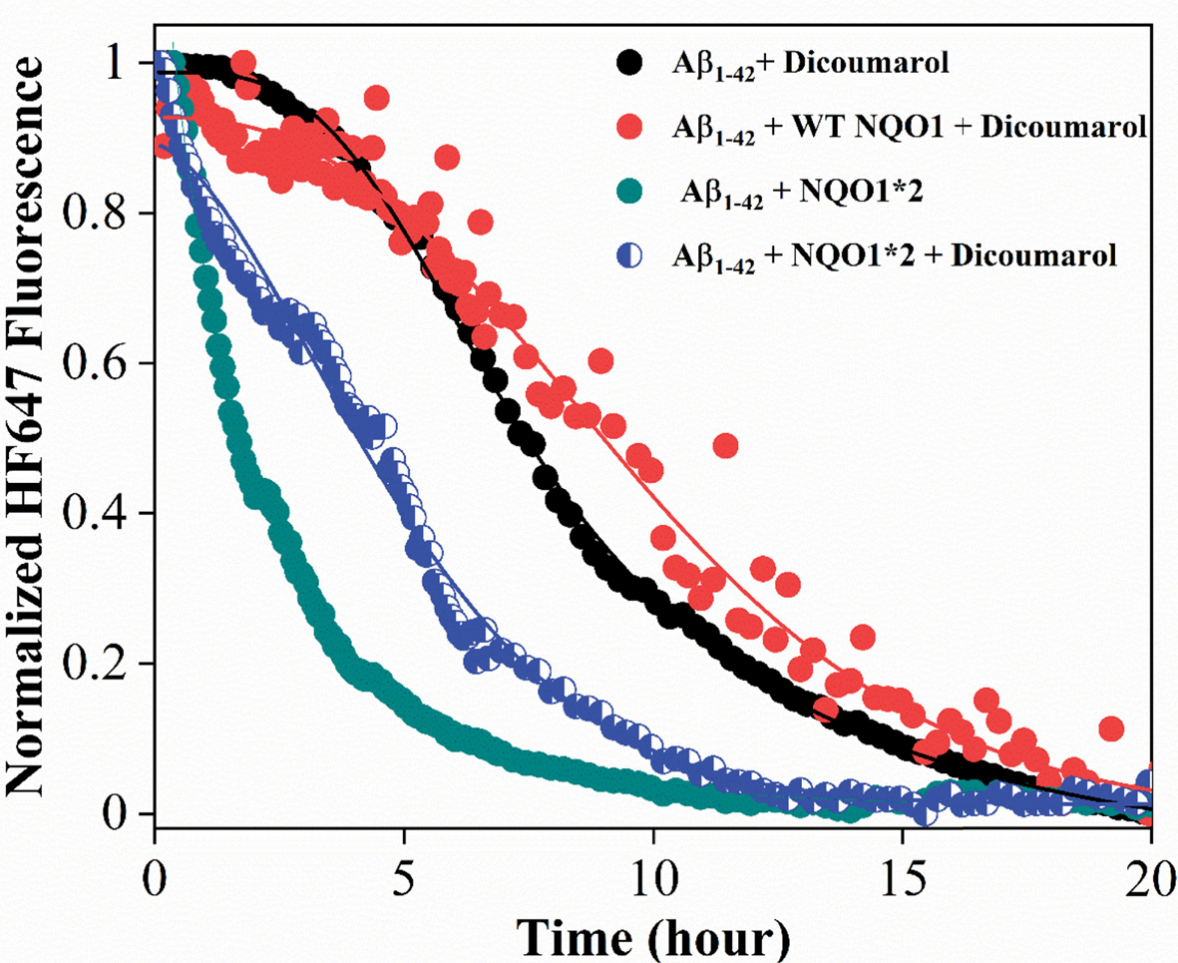
Effect of dicoumarol (20 μM) on Aβ_1-42_ aggregation (10 μM) kinetics in the presence of WT-NQO1 (10 μM) and NQO1*2 (10 μM) in 50 mM phosphate buffer, pH 7.4 at 37°C under atmospheric condition

Dicoumarol binds to NQO1 near the substrate/NADPH-binding site (Figure 1) and has been shown to stabilize NQO1*2 [16]. Figure 5 shows the effect of dicoumarol on the aggregation kinetics of Aβ_1-42_ when monitored using HF647 fluorescence in the presence of WT-NQO1 and NQO1*2. No change in aggregation kinetics was observed in the presence of WT-NQO1 and dicoumarol (red curve in Figure 5) compared with Aβ_1-42_ alone (black curve in Figure 5), similar to the control experiments in Figure 4 in the absence of dicoumarol. However, NQO1*2-induced Aβ_1-42_ aggregation kinetics were markedly slower in the presence of dicoumarol (blue curve in Figure 5) as shown by the prolonged time period to form mature amyloid fibrils. The data clearly demonstrate that stabilizing FAD-deficient NQO1*2 with small molecules such as dicoumarol decrease NQO1*2-induced Aβ_1-42_ aggregation.

### Aβ_1-42_ coaggregates with NQO1-forming larger aggregates with different morphology

**Figure 6:**
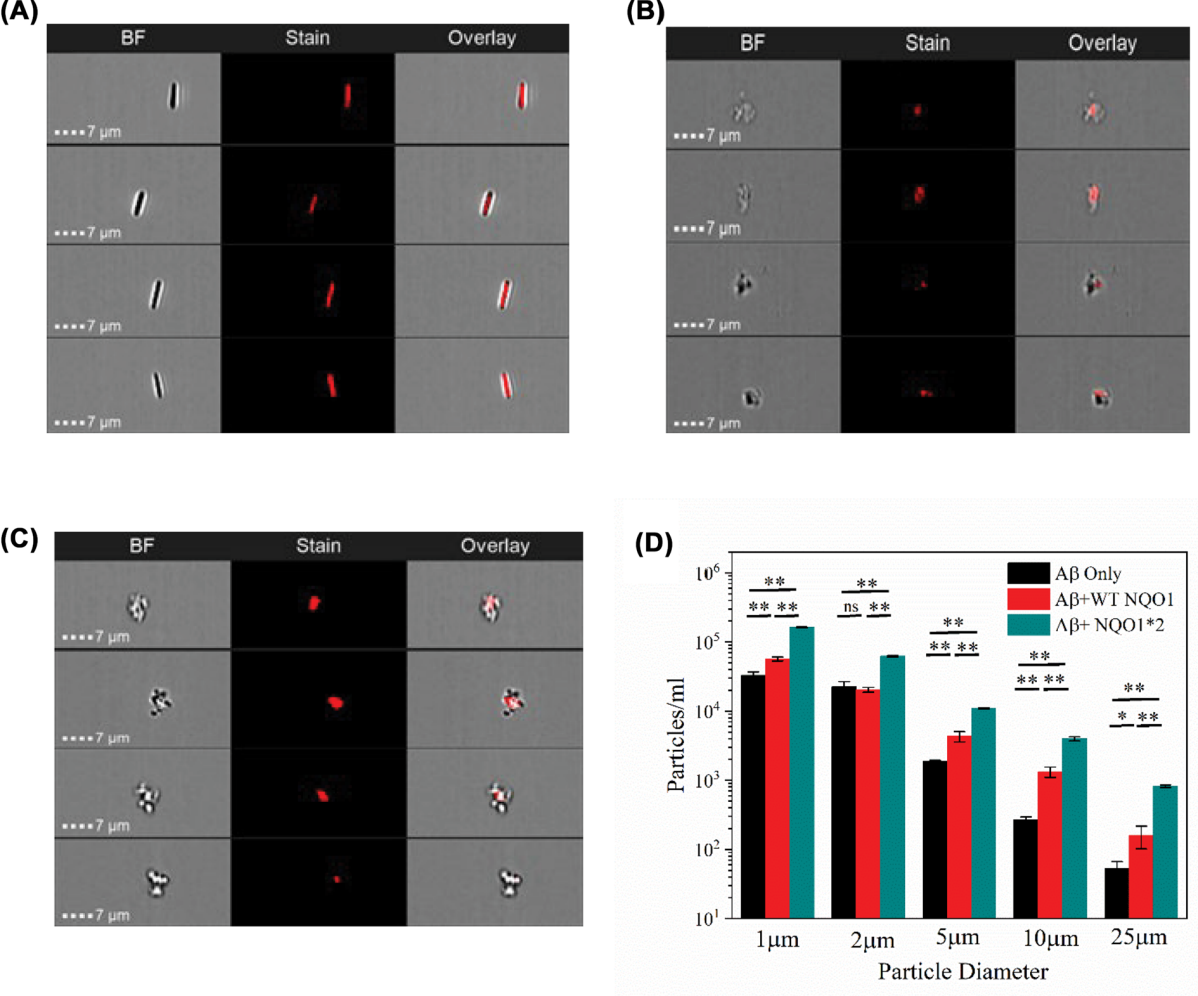
Coaggregation of NQO1 isoforms and Aβ_1-42_ Imaging flow cytometry images of (**A**) Aβ_1-42_ alone, (**B**) Aβ_1-42_ + WT-NQO1, and (**C**) Aβ_1-42_ + NQO1*2. BF represents the BF images, stain represents the total fluorescence images of Aβ_1-42_ covalently tagged with HF647 dye. Combination of BF and stain images are presented in overlay images. (**D**) Particle diameter distribution of Aβ_1-42_ in the absence and presence of WT NQO1 and NQO1*2 as measured by FlowCam technique. The data are presented as the means ± SE from three experiments. One-way ANOVA was used for the statistical comparison. **P<0.01, *P<0.05. Abbreviation: ns, not significant.

Aβ_1–42_ forms unique characteristic mature fibrils after aggregation. We have used the highly sensitive fluorescence imaging flow cytometer Amnis ImageStreamX Mk II (ISX) to characterize the morphology of aggregated Aβ_1–42_ fibrils formed in the absence and presence of WT-NQO1 and NQO1*2 using HF647 fluorescence (Figure 6). Since only Aβ_1–42_ is covalently tagged with HF647, no fluorescence is observed from NQO1 present in the aggregates and is only observed in brightfield (BF) images. As expected, Aβ_1–42_ alone forms rod-shaped mature fibrils (Figure 6A). However, in the presence of WT-NQO1 (Figure 6B) or NQO1*2 (Figure 6C), the aggregates were much larger and more globular with the center mainly composed of red fluorescent Aβ_1–42_ surrounded by unlabeled NQO1 molecules as can be seen from the overlay of BF and fluorescence images. To further characterize the aggregates, FlowCam was used to obtain the size distribution of particles (Figure 6D). Although the size distribution was similar despite differing morphologies between Aβ_1–42_ alone and in the presence of NQO1 isoforms, the greatest number of aggregates were detected using NQO1*2 (Figure 6D). Because the aggregates of Aβ_1–42_ alone and in the presence of NQO1 isoforms show distinct morphologies (Figure 6A–C), particle diameters estimated by FlowCam are in general weighed more toward the major axis if the particle shape becomes ellipsoid or rod shaped.

### NQO1 monomer binds stoichiometrically to Aβ_1–42_

**Figure 7:**
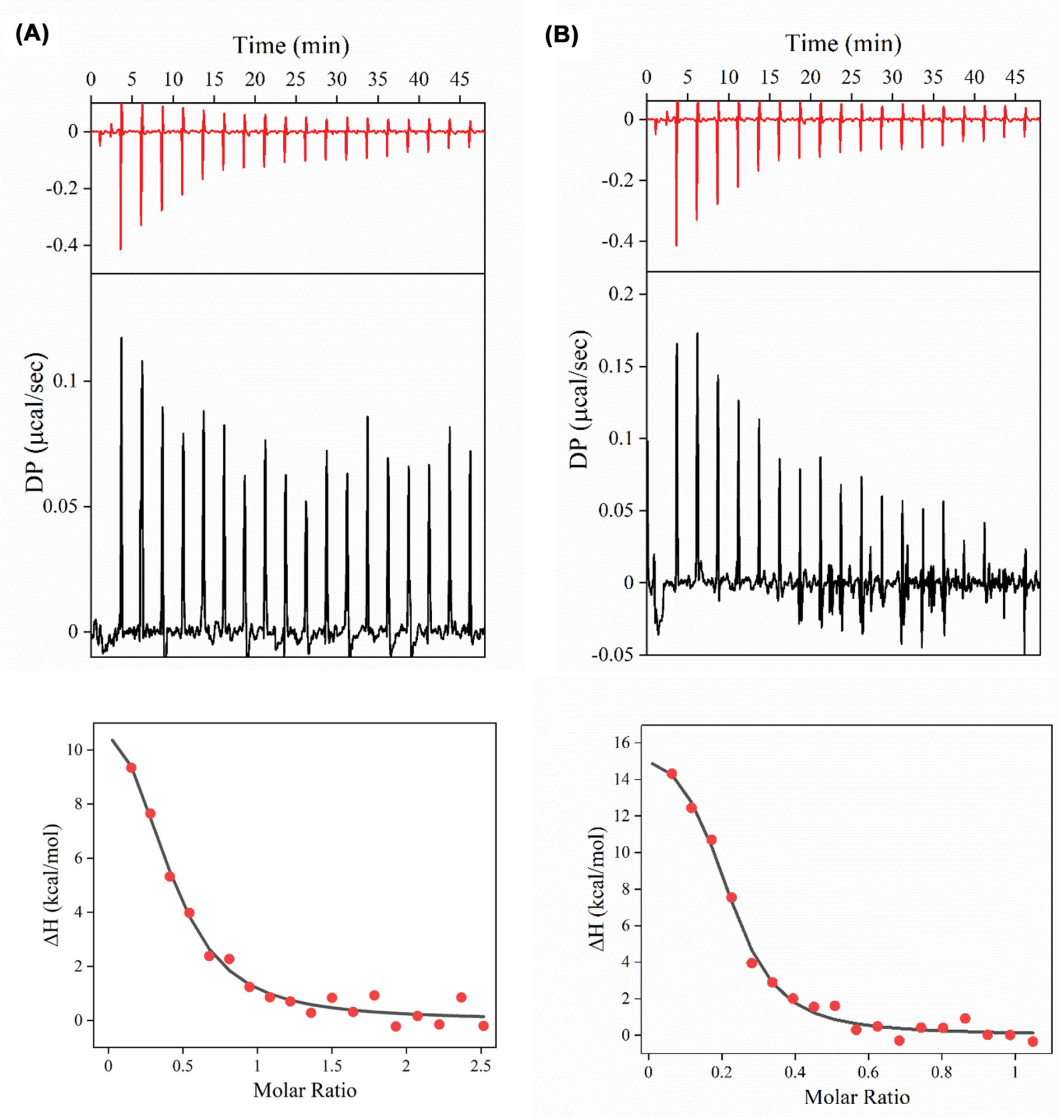
Binding of NQO1 isoforms to Aβ_1-42_ monomer ITC studies of (**A**) WT-NQO1 and (**B**) NQO1*2 binding to Aβ_1-42_ monomer. The upper two panels show the raw heats observed with successive injections from the syringe containing Aβ_1-42_ into the bulb with buffer and with NQO1 isoforms. The lower panel shows the integrated data obtained after subtracting the heat of dilution from the buffer. Data fitting was performed with the nonlinear regression analyses of one site-binding model. The obtained average thermodynamic parameters from three measurement are: (a) *K*_d_ = 1.6 ± 1.0 μM, *N* = 0.4 ± 0.2, Δ*H* = 11.2 ± 2.8 kcal/mol, and Δ*S* = 62.5 ± 9.2 cal/mol.deg; (b) *K*_d_ = 0.4 ± 0.1 μM, *N* = 0.3 ± 0.1, Δ*H* = 15.7 ± 0.8 kcal/mol, and Δ*S* = 79.7± 2.8 cal/mol.deg.

To determine the nature of interaction between Aβ_1–42_ and NQO1 isoforms, binding experiments were performed using ITC. Both WT-NQO1 and NQO1*2 resulted in an endothermic reaction when titrated with monomeric Aβ_1−42_ (Figure 7A,B). Thermograms obtained from integrated heat changes from successive titrations were fitted to a one site-binding model after subtracting from the respective buffer blanks (Figure 7A & B, bottom panels). Both WT-NQO1 and NQO1*2 bind to Aβ_1−42_ with a similar stoichiometry (*N*∼0.3), excluding the possibility of a nonspecific interaction. NQO1*2 binds to Aβ_1−42_ with a higher affinity (*K*_d_ = 0.4 ± 0.1 μM) than that of WT-NQO1 (*K*_d_ = 1.6 ± 1.0 μM), which correlates with the observation that Aβ_1−42_ accelerates coaggregation kinetics of NQO1*2 at a faster rate compared with WT-NQO1 (Figures 2–4). In addition, changes in enthalpy and entropy are positive (values included in Figure 7 legend), indicating that the interaction between Aβ_1−42_ and NQO1 isoforms is hydrophobic in nature [17,33].

## Discussion

The flavoproteome is an important group of proteins in terms of human health and most of the approximately 100 proteins participate in critical metabolic pathways while mutations in a large proportion of flavoproteins result in disease [15]. Mutations in flavoproteins can adversely affect the binding of FAD leading to proteasomal degradation [2,34]. FAD-deficient forms of flavoproteins either as a result of mutation or riboflavin deficiency can result in proteasomal overload and subsequent interaction of FAD-deficient proteins with other proteins can lead to aggregation [18,35]. Using NQO1 as a model flavoprotein and using animal and cellular models, FAD-deficient forms of NQO1, as a result of either riboflavin limitation or the NQO1*2 mutation (P187S mutation in WT-NQO1), were shown to result in β-amyloid aggregation potentially contributing to protein aggregation diseases [18]. However, these earlier studies did not investigate a potential direct interaction between Aβ_1-42_ and NQO1 isoforms, and the nature of any such interaction. In this study, we have characterized the kinetics of Aβ_1-42_ aggregation in the presence of NQO1*2 in detail and defined the shape and multiplicity of NQO1/Aβ_1-42_ aggregates.

In fluorescence studies using either ThT or the amyloid-specific probe HF647, aggregation of Aβ_1-42_ alone could be observed that was increased by the presence of NQO1 isoforms. FAD-deficient forms of NQO1, NQO*2 that binds to FAD with a much lower affinity compared with WT-NQO1 [17] or the apo-form of NQO1 that completely lacks bound FAD, led to marked accelerated aggregation of Aβ_1-42_ relative to WT-NQO1 with a much-shorter lag phase. Experiments under a nitrogen atmosphere rather than aerobic conditions excluded the possibility that NQO1 aggregation serves as a nucleation trigger for amyloid aggregation suggesting interactions between monomeric NQO1 and Aβ_1-42_ monomer, which was confirmed using ITC.

The NQO1*2 protein is considerably less stable in cells than WT-NQO1 and undergoes rapid proteasomal degradation [1,36]. The NQO1*2 protein is more prone to unfolding than WT-NQO1 and has a markedly lower affinity for FAD than WT-NQO1 [17,22,37,38]. The NQO1*2 mutation results in partial unfolding of the terminal CTD [18], but its effects also propagate throughout the protein structure [37,39]. Dicoumarol has been shown to stabilize the dynamic CTD of FAD-deficient isoforms of NQO1 and to rescue the instability of the NQO1*2 protein in cellular systems [17,40]. In our experiments, dicoumarol inhibited the aggregation of Aβ_1-42_ in the presence of the NQO1*2 protein confirming its role as a stabilizing pharmacological chaperone of NQO1 [40]. We suggest that the dynamic CTD of NQO1*2 may play a role in its interaction with Aβ_1-42._

The shape of the aggregates formed between NQO1 and Aβ_1-42_ was investigated using fluorescence flow cytometry. Aggregation of Aβ_1-42_ alone generated rod-shaped fibrils as expected, but aggregates in the presence of either WT NQO1 or NQO1*2 resulted in globular aggregates with a center of Aβ_1-42_ surrounded by NQO1 molecules (Figure 6), since only Aβ_1-42_ is labeled with HF647 and NQO1 isoforms are unlabeled. Particle size distribution analysis showed the highest number of aggregates were formed in the presence of FAD-deficient NQO1*2 relative to WT-NQO1 or Aβ_1-42_ alone. ITC analysis indicated that NQO1 isoforms bound stoichiometrically to Aβ_1-42_ driven by hydrophobic interactions with NQO1*2 binding to Aβ_1-42_ with a higher affinity than WT-NQO1.

FAD-deficient proteins leading to enhanced aggregation and proteasomal overload can potentially impact the triggering of neurodegenerative diseases [18]. Accordingly, supplementing with flavin mononucleotide (FMN), a precursor of FAD, decreased misfolded protein load, decreased Aβ_1-42_ toxicity as well as the toxicity of other proteins inducing aggregation-driven diseases, such as HTT huntingtin protein and α-synuclein, and demonstrated beneficial effects on proteostasis [35]. In addition, FAD-deficient NQO1 isoforms have an extended interactome with other proteins including proteostasis network components. WT-NQO1 is known to interact with other proteins but its interactome was substantially larger under FAD restriction as was the interactome of the NQO1*2 protein due to its FAD-deficiency, instability, and disordered structure [41]. The significance of these observations is that the FAD-deficient proteins can play an important role in the mechanism of protein aggregation by extending their interactions to a broad network of proteins.

Oxidative stress has been associated with amyloidogenesis and AD [42]. NQO1 has also been associated with early pathological changes in AD and its expression correlates with the progression and localization of AD pathology in human brains [6–8]. NQO1 is increased by oxidative stress as a part of the Nrf2 battery of stress response genes and the elevation of NQO1 associated with AD pathology is commonly viewed as a neuroprotective response to the oxidative stress that accompanies AD [6–8]. Interestingly, riboflavin is a neuroprotective agent known to limit oxidative stress, mitochondrial dysfunction, and neuroinflammation [43,44] and to slow the rate of cognitive decline in the elderly [45,46].

In this manuscript, we investigated the role of NQO1 in the aggregation kinetics of Aβ_1-42_ and found that FAD-deficient forms of NQO1 led to accelerated aggregation. The significance of the aggregates we have identified for either toxicity or amyloidogenesis remains to be established. We found that coaggregates of NQO1 and Aβ_1-42_ consisted of an amyloid core unit surrounded by NQO1 molecules. Whether larger, more globular Aβ_1-42_ aggregates formed preferentially in the presence of FAD-deficient NQO1 are more toxic to the cell than rod-like amyloid fibrils remains to be determined in cellular systems. Similarly, whether unstable FAD-deficient forms of NQO1 or other flavoproteins are associated with increased amyloidogenesis *in vivo*, which can be modulated by riboflavin, its precursors, or by stabilizing pharmacological chaperones will need to be tested in animal models. Understanding these links may contribute to further insights into the role of flavoproteins in disease and may also lead to potential novel therapeutic options to limit amyloidogenesis.

## Data Availability

All the data are contained within the article.

## Abbreviations

Aβ_1-42_: amyloid-β peptide
AD: Alzheimer’s disease
BF: brightfield
CTD: C-terminal domain
FAD: flavin adenine dinucleotide
FMN: flavin mononucleotide
HF647: HiLyte Fluor 647
ITC: isothermal titration calorimetry
MFI: microflow imaging
NQO1: NAD(P)H quinone oxidoreductase 1
NTD: N-terminal domain
PMRS: plasma membrane redox system
ThT: Thioflavin T
